# Editorial: Mental health challenges in long-term pharmacotherapy for patients with chronic diseases

**DOI:** 10.3389/fpsyt.2026.1837024

**Published:** 2026-04-10

**Authors:** Zhao Yin, Shangfeng Tang, Xuejun Yin, Shusen Sun

**Affiliations:** 1 Department of Pharmacy, First Affiliated Hospital of Zhengzhou University, Zhengzhou, China; 2 School of Medicine and Health Management, Huazhong University of Science and Technology, Wuhan, China; 3 School of Population Medicine and Public Health, Chinese Academy of Medical Sciences and Peking Union Medical College, Beijing, China; 4 College of Pharmacy and Health Sciences, Western New England University, Springfield, MA, United States

**Keywords:** chronic diseases, health literacy, medication adherence, mental health, pharmacotherapy, quality of life

## Introduction

Chronic diseases represent a dominant global health burden and frequently require long-term or lifelong pharmacotherapy (1). While advances in pharmacological treatments have significantly improved survival and disease control, they have also introduced complex challenges related to patients’ mental health. Increasing evidence suggests that psychological distress—including depression, anxiety, fatigue, and cognitive impairment—is highly prevalent among individuals undergoing long-term pharmacotherapy and plays a critical role in determining treatment adherence, quality of life, and overall clinical outcomes (2).

This Research Topic, “Mental Health Challenges in Long-term Pharmacotherapy for Patients with Chronic Diseases,” brings together 15 articles that explore these issues across diverse disease settings and populations. These contributions provide a comprehensive understanding of the psychological burden associated with chronic disease management and highlight emerging mechanisms and intervention strategies.

## Mental health burden across chronic disease populations

A consistent finding across this Research Topic is the substantial and multifaceted psychological burden experienced by patients with chronic diseases. Depression and anxiety are prevalent across a wide range of conditions, including cancer, infectious diseases, cardiovascular disorders, and metabolic diseases. For example, Lin et al. demonstrated that depression in individuals with lung cancer has become a major research focus because of its significant impact on quality of life and clinical outcomes. Similarly, Lei et al. reported that older patients with tuberculosis exhibit varying levels of anxiety, depression, and insomnia depending on inflammatory and drug-induced liver injury phenotypes, suggesting a biological basis for psychological variability. In the ophthalmologic population, Zhang et al. identified a notable prevalence of anxiety among older adults with cataracts, which was influenced by socioeconomic and health-related factors. At the population level, Li et al. revealed that depression severity is closely associated with an increased risk of chronic diseases and poorer cognitive and life satisfaction outcomes, particularly in individuals with comorbid conditions. Wang et al. demonstrated that depressive symptoms may predict the future development of chronic liver diseases, further emphasizing the bidirectional relationship between mental and physical health.

## Psychological mechanisms and mediating pathways

Beyond prevalence, several studies have elucidated the psychological mechanisms that mediate the relationship between chronic diseases and mental health outcomes. Constructs such as psychological resilience, cognitive flexibility, psychological capital, and basic psychological needs have emerged as critical determinants. Zhao et al. demonstrated that psychological resilience and cognitive flexibility are strongly associated with post-traumatic growth in patients undergoing intensive treatments, such as continuous renal replacement therapy. In oncology settings, Yan et al. identified psychological capital as a key mediator linking medication adherence and cancer-related fatigue, suggesting that enhancing patients’ internal psychological resources may improve both adherence and symptom burden. Similarly, Ding et al. demonstrated that the satisfaction of basic psychological needs influences resilience through pathways involving family support and hope in patients with acute myocardial infarction. These findings highlight that psychological adaptation is not merely a passive response to illness, but an active and modifiable process shaped by internal and external resources.

## Medication adherence, behavior, and health literacy

Medication adherence is a cornerstone of effective long-term pharmacotherapy and is closely intertwined with patients’ mental health. Psychological distress can impair adherence, and poor adherence may worsen disease outcomes, creating a self-reinforcing cycle. Sun et al. demonstrated that medication behavior among children and adolescents is significantly influenced by perceived stress, health literacy, and self-efficacy. Griese et al. showed that individuals with chronic mental illness have lower health literacy than those with somatic conditions, highlighting an important vulnerability in managing long-term treatment. These findings emphasize that adherence should be viewed not solely as a behavioral issue, but as a multidimensional construct influenced by psychological, cognitive, and social factors. Addressing these determinants is essential for optimizing therapeutic outcomes.

## Impact on quality of life and functional outcomes

Mental health challenges have profound implications for patients’ quality of life and functional status. Several studies in this Research Topic identified key pathways linking psychological factors to patient-reported outcomes. Zheng et al. demonstrated that well-being mediates the relationship between fatigue and health-related quality of life in patients with chronic hepatitis B, suggesting that interventions targeting well-being may yield broad benefits. Similarly, Li et al. reported a high prevalence of psychotic-like experiences among patients with diabetes, which are strongly associated with impaired quality of life. Cognitive function is another important dimension of the mental health burden. Wang et al. identified sex-specific differences in cognitive impairment among patients with bipolar disorder, highlighting the need for personalized approaches to mental health management.

## Risk factors and vulnerable populations

The studies included in this Research Topic identified a range of risk factors contributing to mental health challenges. These include demographic variables (age, sex, and socioeconomic status), clinical characteristics (disease severity and comorbidities), lifestyle factors (sleep and physical activity), and biological mechanisms (inflammation and genetic polymorphisms). For instance, Zhang et al. highlighted the roles of socioeconomic status, sleep duration, and physical health in shaping anxiety risk among older adults. Jurczak et al. explored the influence of genetic polymorphisms on coping strategies in cancer patients, suggesting a potential biological basis for psychological adaptation. These findings highlight the importance of risk stratification and individualized care approaches in addressing mental health challenges in individuals with chronic diseases.

## Emerging interventions and future directions

This Research Topic also provides insights into potential interventions. Liu et al. demonstrated that traditional Chinese exercise can significantly improve both physical function and mental health outcomes in patients with chronic obstructive pulmonary disease, highlighting the value of non-pharmacological approaches. These studies suggest that the effective management of chronic diseases requires an integrated, multidisciplinary approach that addresses both the physical and psychological dimensions of care. Future research should focus on developing scalable interventions, incorporating mental health assessments into routine clinical practice, and leveraging digital health technologies to enhance patient engagement.

## Conclusion

The articles in this Research Topic highlight the complex and bidirectional relationship between long-term pharmacotherapy and mental health in patients with chronic diseases. Psychological challenges are pervasive and significantly influence treatment adherence, disease progression, and quality of life. Addressing these challenges requires a paradigm shift toward holistic, patient-centered care models that integrate mental health into chronic disease management. By advancing our understanding of these interactions and identifying actionable pathways for intervention, this Research Topic provides a foundation for improving outcomes and enhancing the quality of care for patients worldwide.
